# Cubism and Research Synthesis

**DOI:** 10.3201/eid2102.AC2102

**Published:** 2015-02

**Authors:** Salaam Semaan

**Affiliations:** Centers for Disease Control and Prevention, Atlanta, Georgia, USA

**Keywords:** art science connection, emerging infectious diseases, art and medicine, research synthesis, systematic reviews, metaanalysis, evidence-based public health, evidence-based medicine, Fernand Léger, mechanical elements, cubism and research synthesis, about the cover

**Figure:**
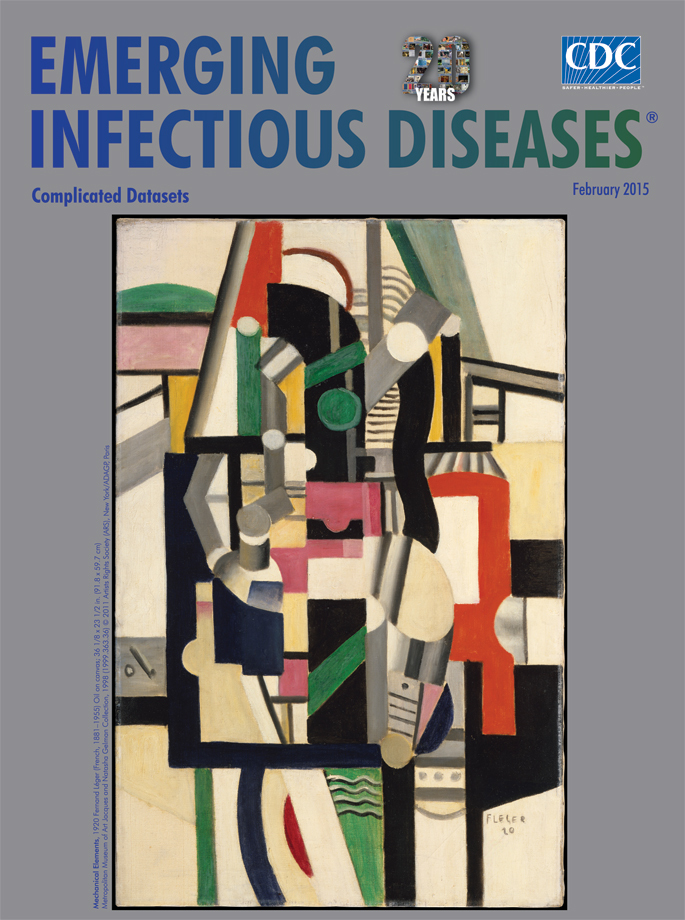
**Fernand Léger (1881–1955) *Mechanical Elements*, 1920.** Oil on canvas; 36 1/8 in × 23 1/2 in (91.8 cm × 59.7 cm) Metropolitan Museum of Art, Jacques and Natasha Gelman Collection, 1998 (1999.363.36) © 2011 Artists Rights Society (ARS), New York/ADAGP, Paris

French artist Fernand Léger (1881–1955) embraced the Cubist art movement of fracturing objects into geometric shapes. The term cubism draws on the art critic Louis Vauxcelles’ reference to the bizarre cubes he saw in budding artwork that fragmented form into interlocking blocks. Many Cubists reduced objects into cylinders, spheres, and cones and painted them in a single plane as if all faces of an object are visible simultaneously or successively. Influenced by his background as an architectural draftsman and by modernism, Léger was interested in the relationship between color and architecture, perhaps to express the noise, dynamism, speed, and movement of new technology and machinery.

Léger’s unique brand of cubism was distinguished by his focus on geometric forms, use of brilliant primary colors, bold black outlines, and belief that everyone could understand art. Léger adapted cubism techniques to break down forms into tubular shapes. His predominant style in 1910 was nicknamed “Tubism.”

Cubism can evoke in our minds the methodology of research synthesis conducted via systematic reviews and meta-analysis. Synthesis of clinical or public health research, conducted by combining results of several studies, explores the relationship between an intervention or an exposure and a health outcome from perspectives different from and in addition to those examined in a single study. In cubism, the artist paints an object from different or successive angles as a single image on a single canvas. In research synthesis, clinical and public health professionals examine the scientific literature on a certain topic from multiple years and perspectives. Meta-analysts display the literature in one publication in different figures, including forest plots, funnel plots, and chronological cumulative meta-analysis.

Cubist artwork creates a feeling of movement and shape that can differ from what an object represents. Research synthesis allows for examining scientific studies of diagnostic tools, therapies, interventions, programs, or policies as part of a continuum, with the past flowing into the present and the present influencing the future. By revolutionizing the way objects are depicted and painted, Cubists created a dialogue among art critics, collectors, dealers, and the general public. Research synthesis creates a dialogue among clinical and public health professionals about its role in evidence-based medicine and public health. Set against a framework of thick, black horizontal and vertical lines, Léger’s *Mechanical Elements* is characterized by cones, cylinders, disks, and parallel wavy lines that can evoke the image of a new machine age and its momentum or a forest plot and the data of many research studies. In their syncopated arrangement, new technologies transform the world. In a synthesized and pooled effect size, meta-analysis provides new implications for research, programs, and policy.

Learning how to conduct and apply systematic reviews and meta-analysis may be similar to learning how to use machinery. One must learn concepts (e.g., elements of research synthesis, the technology), repeat the behaviors (e.g., use relevant software, operate the machine), and practice relentlessly. Just as art can help the public understand history, clinical and public health professionals can use research synthesis as a scientific tool to enhance medicine and public health. To promote evidence-based programs and policies, the primary single-study articles and research synthesis published in this journal can remind us that we can learn from art and science to conduct, value, and apply evidence-based medicine and public health.
